# Regional citrate anticoagulation in high-volume continuous venovenous hemodialysis

**DOI:** 10.1186/cc9548

**Published:** 2011-03-11

**Authors:** R Kalb, J Ammann, T Slowinski, S Morgera, D Kindgen-Milles

**Affiliations:** 1 University Hospital, Düsseldorf, Germany; 2 Charité, Berlin, Germany

## Introduction

Regional citrate anticoagulation (RCA) is a new anti-coagulation mode for continuous renal replacement therapy (CRRT). Compared with heparin anticoagulation, RCA prolongs filter lifetime, decreases transfusion requirements, and yields good metabolic control [1,2]. However, RCA was not investigated in patients requiring dialysis doses of >3 l/hour because of severe metabolic derangements or obesity. We investigated whether RCA for CVVHD is safe and effective also in patients in need of such intensified treatment. We focused on the filter lifetime, delivered dialysis dose, and control of acid-base balance.

## Methods

In a prospective observational study we enrolled 75 patients with acute kidney failure (AKF) following extended surgery. High-volume CVVHD was applied using RCA for at least 72 hours. Minimum dialysis dose was targeted at 45 ml/kg/hour. According to the protocol, for effective anticoagulation, a citrate dose of 4 mmol/l blood and a calcium infusion of 1.7 mmol/l dialysate was required. We measured arterial blood gases and levels of ionized calcium pre-filter and post-filter every 4 hours. Blood flow, dialysis dose and doses of citrate and calcium were registered as well as filter lifetime and the reason for downtime.

## Results

The mean dialysis dose during the first 72 hours of treatment was 49 ± 14 ml/kg/hour, corresponding to a dialysate flow of 3,736 ± 88 ml/hour. Mean blood flow was 177 ± 4 ml/minute. The mean citrate dose applied during the first 72 hours was 3.83 ± 0.07 mmol/l. The mean calcium dose was 1.85 ± 0.06 mmol/l. Severe hypocalcemia/hypercalcemia did not occur. In one case an increasing demand for calcium substitution occurred after 84 hours that was indicative of citrate accumulation but the total/ionized calcium index was never higher than 2.5. After 72 hours of CVVHD, acidosis (pH <7.35) occurred in 7% (5/75) of all patients, an alkalosis (pH >7.45) in 22% (16/73) while 71% (52/73) showed a normal pH. Mean filter lifetime was 78 ± 2 hours. Thirteen treatments were stopped because of filter clotting, in all the remaining 87 filters stopping of treatment was caused by other reasons (surgery, diagnostic procedures, restored diuresis, death). There were no bleeding complications related to renal replacement therapy. In-hospital mortality was 57% (43/75).

## Conclusions

Regional citrate anticoagulation for CVVHD is safe and effective to deliver a high dialysis dose, to control acid-base status, and to yield excellent filter lifetimes in postoperative AKF.
